# A ‘forgettable minority’? Psychiatric Institutions and the Intellectually Disabled in Ireland, 1965–84

**DOI:** 10.1093/shm/hkaa015

**Published:** 2020-05-25

**Authors:** David Kilgannon

**Keywords:** Irish history, disability history, institutional history, psychiatric history

## Abstract

This article investigates the admission of the intellectually disabled to institutional psychiatric facilities in the Republic of Ireland between 1965 and 1984, using this as a way to explore disability provision and the later years of the state’s congregate mental hospital network. Drawing on institutional documents and news media, it argues that ‘handicap admissions’ continued along an established pattern, while demonstrating how these facilities remained ill-equipped to meet the needs of disabled residents. In doing so, this article begins to address the broader lacuna surrounding intellectual disability within Irish historiography, while complicating an emergent body of work on the ‘deinstitutionalisation’ of the state’s psychiatric hospitals during the late twentieth century. It suggests ways in which institutional records can be used to access patient experiences and highlights the need for further research on intellectual disability, examinations of which can contribute towards the histories of institutionalisation and social policy in post-war Ireland.

Speaking in 1982, journalist Hilary Orpen presented a scathing critique of ‘handicap wards’ within Irish psychiatric institutions.^1^ ‘To enter here is like a descent into hell’ was her assessment of St. Brigid’s Hospital in Ballinasloe, which she visited for *Today Tonight*, a current affairs programme on the national broadcaster RTÉ (Raidió Teilifís Éireann). Her narration was superimposed over footage of slumped bodies, stained walls and sparsely furnished wards, where ‘the dark and squalid accommodation reeks of excrement. There is no activity, no communication. The Mentally Handicapped sit and rack and moan. They are forgotten people, abandoned here for life’.^2^ Although it was reminiscent of a prison, Orpen emphasised that the ‘people incarcerated here have committed no crime’, they resided in these conditions due to a ‘less than average intelligence’.^3^ The tone of this report, and the surrounding press coverage, underscored how life in St. Brigid’s was regarded as unacceptable.^4^ Yet, institutional reports point to (equally deficient) analogues of this ‘handicap ward’ within hospitals across the country, as in 1981 there were 2,170 residents in psychiatric care with a primary diagnosis of intellectual disability, which represented 15.5 per cent of in-patients.^5^

In line with international trends, the latter decades of the twentieth century were characterised by decline across congregate psychiatric institutions in Ireland, as out-patient facilities and psychopharmacological interventions contributed towards a (seemingly) inexorable drop in their resident populations.^6^ Although correct, this narrative of institutional decline neglects those left behind within the remaining ‘mental hospital’ infrastructure. Indeed, historical research on post-war psychiatric care remains notably underdeveloped in Ireland, despite a range of ‘asylum studies’ that explore institutional provision throughout the nineteenth- and early twentieth centuries.^7^ There is a limited discussion of post-war trends in Damien Brennan’s *Irish Insanity*, while Brendan Kelly’s history of psychiatry offers a similarly concise summary before arguing that ‘it is still too early to present valid historical evaluations of the contributions’ made by the era’s physicians and policymakers.^8^ This reluctance to explore recent history contrasts against a growing body of British research, which has examined changes to institutional practices into the late twentieth century.^9^ Vicki Long, for instance, has addressed these developments in Scotland, describing how the steady growth in community-based psychiatric services did not ‘rapidly eradicate institutional care’ but instead caused residential services to evolve in response to changing patient demographics and the rise of ‘care in the community’ within statutory policy.^10^

This article explores the admission of the intellectually disabled to institutional psychiatric facilities in the Republic of Ireland between 1965 and 1984; a period, from the publication of the *Report of the Commission on Mental Handicap* to the *Towards a Full Life* green paper on disability, which was marked by a sustained decline in Ireland’s psychiatric inpatient population and the persistence of ‘handicap wards’ in hospitals nationwide. It examines both why these ‘handicap admissions’ continued and the experiences of the intellectually disabled within these institutions during the late twentieth century. In doing so, it begins to address the confluence of two under-examined areas within the state’s historiography as, alongside the paucity of work on post-war psychiatry, disability history is a relatively new area of enquiry and there is a lack of research on the ‘mentally handicapped’ in Ireland.^11^ Drawing on a diverse range of institutional documents and news media, this article uses hospital records to explore the experiences of the disabled resident. This is by no means a new approach within the history of medicine. Jonathan Andrews has demonstrated how institutional documents, like patient case notes, ‘may provide the surest basis we have for understanding the changing nature of the experiences of the insane in asylums’ during the nineteenth century.^12^ Subsequent work on institutional life has built on this approach, showing the extent to which a range of institutional sources can reveal the experiences of patients in a congregate environment.^13^ To be clear, an analysis of hospital records can never hope to replicate the level of complexity within a disabled person’s ‘life history’.^14^ Nonetheless, in the absence of such accounts, institutional records can begin to provide an insight into how post-war Irish psychiatric institutions functioned in practice, thus beginning to ‘lower the historical gaze onto the sufferers’ who lived within these facilities.^15^ This analysis spotlights a gap between policy goals and their implementation in centres across the country, showing how deficient living conditions on physically ailing wards shaped the lives of disabled residents. After positioning ‘handicap admissions’ along a broader chronology, the article explores how the disabled entered these facilities during the late twentieth century, before addressing the nature of life within ‘handicap wards’. This article contends that by understanding the persistence of ‘handicap admissions’, historians can uncover the broader landscape of provision for the intellectually disabled across the twentieth century, while also complicating the underdeveloped history of psychiatric services in post-war Ireland. Existing work on deinstitutionalisation has focused on policy developments and the statistical decline of the ‘mental hospital’ population.^16^ By moving beyond this approach, we can begin to understand the history of the intellectually disabled within psychiatric institutions, a narrative that complicates the decline of these institutions by showing the long-standing persistence of their ‘ancillary’ role in accommodating the ‘mentally handicapped’.

## Established Practices: ‘Handicap Admissions’ 1833–1965

The Victorian-era asylum housed a range of needy residents, as they were founded to ‘clear the roads of wandering idiots and the gaols and houses of industry of troublesome lunatics’.^17^ This goal was reflected in the 1843 rules for the operation of asylums, and in legislation like the 1845 Lunacy Act, both of which included acute mental illness and intellectual disability under the umbrella of ‘lunacy’.^18^ This broad definition, as well as the limited number of residential beds in disability-specific institutions, meant that the ‘mentally handicapped’ were admitted to asylum facilities as part of the broader ‘institutional marketplace’ throughout the nineteenth century.^19^ Indeed, this pattern was already established in 1833, when an assistant commissioner for Whateley’s Commission (the Royal Commission on the Poorer Classes in Ireland) visited the Connacht District Lunatic Asylum in Ballinasloe, Galway. The Commissioner opposed plans to expand the existing facility, which would later become St. Brigid’s psychiatric hospital, as it already housed large numbers of the congenitally disabled. Further expansion was therefore not advisable, he argued, as the state was simply ‘erecting palaces for the permanent accommodation of [Ireland’s] slavering and worthless idiots’.^20^ Regardless of their considerable scale, these Victorian institutions struggled to address persistent calls to admit ‘incurable patients’, like the intellectually disabled or those with epilepsy, to their already overcrowded wards.^21^ In her study of institutional management in the South-East, Catherine Cox described how local committees were increasingly concerned by the entry of those with long-term needs to asylums; a practice that, a contemporary observer noted, had precipitated the degeneration of some centres into ‘domiciles for incurable lunatics to the exclusion and serious detriment of acute cases’.^22^ Political independence in 1922 did little to shift this entrenched practice, as the 1927 *Commission on the Relief of the Sick & Destitute Poor* noted that the newly renamed District Mental Hospitals continued to accommodate a range of residents beyond those with an acute mental illness.^23^

Mark Finnane argued that broader ‘political conditions in Ireland precluded the serious consideration of the status of the insane asylum’ throughout the nineteenth century.^24^ This failure to engage with psychiatric provision was sustained during the early years of the state, until the first substantive attempt at reform with the 1945 Mental Treatment Act. This legislation replaced the 1867 Dangerous Lunatics Act and initiated a range of reforms to the state’s mental hospitals, including changes to terminology (‘patient’ instead of ‘inmate’), expanding out-patient provision and creating a voluntary hospital admissions process.^25^ It also introduced a clear shift in administrative power. Previously, a committal to the institution involved magistrates (later peace commissioners) who authorised a doctor’s certificate of admission. Under the new regulations, admission was made on foot of a clinical decision alone.^26^ This represented a consolidation of medical influence, as doctors now had the sole authority to determine who resided in their hospital.^27^

Yet, without an alternative form of care for the intellectually disabled, doctors remained compelled to continue admitting ‘incurables’ to their facilities throughout the mid-twentieth century. The newly founded Department of Health was aware of this practice, as the National Inspector for Mental Hospitals repeatedly noted the sizeable ‘handicap’ patient population within his annual surveys of the psychiatric system.^28^ In his memoir *Music and Madness* (2008), Dr Ivor Browne, former chief psychiatrist of the Eastern Health Board, discussed the broad role that these institutions played throughout the mid-century. For Browne, the Irish ‘mental hospital’ system that he joined as a junior doctor in 1957 was not just a curative institution for those with acute psychiatric illnesses, but also served as a long-term residence for a heterogenous population of ‘those for whom society had no place [and] are dumped to get them out of sight - the aged, the mentally retarded, the epileptic, the disturbed’.^29^ The Victorian-era admission of ‘incurables’ had obviously persisted into the 1950s. Indeed, while anecdotal, there is varied evidence of psychiatric hospitals retaining their role as housing for potentially ‘troublesome’ individuals, away from wider society. A nurse, who worked at St. Columba’s hospital in Sligo, described how ‘it was no secret that many that went to St. Columba’s were social cases - an old person or someone the family couldn’t get on with’.^30^ Similarly, Dr Maurice Guéret recalled how his grandfather, Resident Medical Superintendent (RMS) of the Central Mental Hospital in Dundrum, was compelled to admit the notorious abortion provider Mary Anne ‘Mamie’ Cadden in the 1950s, despite the clinically inconvenient fact that ‘she had no mental illness’.^31^ Thus, the mid-century psychiatric hospital retained its long-standing ancillary role as accommodation for a miscellaneous population that commonly included ‘the poor, the eccentric, the socially troublesome, the vulnerable and the unwanted’.^32^

This reliance on local ‘mental hospitals’ was augmented by public demand for (oversubscribed) ‘handicap’ institutions. Hospital committee minutes repeatedly underline the reluctance of governing boards to admit ‘Mental Defectives’ to their institutions, but note the pressure to do so due to a lack of alternative forms of secure accommodation. The minutes of a joint meeting of Grangegorman and Portrane Mental Hospital Boards in June 1948 acknowledged how their facilities housed 12 patients under the age of 16 years and that ‘all these cases are suffering from Mental Deficiency of varying grades, from idiocy to imbecility’.^33^ The 1949 committee minutes from Grangegorman Mental Hospital similarly underlined the role of the facility in providing secure institutional accommodation for vulnerable disabled children, as the RMS stated that:

There are six mental defectives seven years of age or under and nine between the ages of 8 and 15 … All are suffering from low-grade mental deficiency, some with epilepsy, some with complete lack of control of natural habits and some extremely mischievous and restless. These little patients had to be admitted because it was represented that there was no alternative accommodation and no means of managing them in their home. The only way in which these children could be treated in this Hospital was by scattering them through the adult wards.^34^

Nationwide, 15 children under the age of 10 years, and 33 between the ages of 10 and 14 years, were placed in district mental hospitals during 1956 alone, the majority of whom were admitted due to a congenital intellectual disability.^35^ Examining mid-century records from Grangegorman Hospital in Dublin, Fiachra Byrne has posited that the institution served as a form of emergency accommodation; used when care in the home was no longer possible for a disabled child with behavioural challenges, the hospital acted as an emergency ‘salve to conflicts within families’.^36^ Indeed, the 1956 Inspector of Mental Hospital’s report even discussed plans to formalise the continued practice of admitting severely ‘handicapped’ children to St. Ita’s Hospital in Portrane, North Dublin, as he noted the hospital’s proposal to establish a designated unit for their population of ‘disturbed juvenile mentally handicapped’ patients.^37^

Providing specific examples of these ‘handicap admissions’ remains challenging, both due to the inaccessibility of contemporary records and the changing nature of diagnostic categories over time.^38^ Yet, the unusually detailed record of one young boy’s experience demonstrates the continued use of psychiatric hospitals as a venue to house the intellectually disabled person with ‘challenging’ behaviour. Described as a ‘mute child’, Gerard was 7 years when admitted to the Limerick District Mental Hospital in 1946.^39^ The Hospital’s RMS sought the National Inspector’s advice about the boy’s care, providing a detailed history of Gerard’s previous accommodation. He originally entered his local County Home in Limerick, before being sent to the St. Vincent’s home in Dublin, a specialist institution for the intellectually disabled operated by the Daughters of Charity of Saint Vincent De Paul. He was soon sent back to the county home in Limerick due to complaints about his ‘mischievous’ behaviour. The county home also experienced trouble with Gerard, as he was ‘violent and bites and spits when restrained and attempted to get through windows’.^40^ Due to this, Gerard was sent to his local psychiatric hospital.

The doctor’s concern for this young boy was obvious. He detailed an effort to send the child elsewhere as he ‘tried to have him admitted to the Stuart [*sic*] institute and also to St. Augustine’s colony Blackrock, but both were unable to receive him’.^41^ The Inspector’s reply provides some insight into the unexceptional nature of this situation. While he recommended that the doctor continue to contact specialist institutions, he also noted that, until Gerard could be sent elsewhere, he should be accommodated on an appropriate ward within the hospital. While the practice was understood as obviously undesirable, particularly for a disabled child, it was used in the absence of an alternative form of secure accommodation.^42^ For a child like Gerard, described as too ‘troublesome’ for either a County Home or a specialist institution, there were simply no other options. This form of ‘handicap admission’ was framed as an intractable problem by Dr K.G. McColgan Barry in the *Kilkenny People* in 1957, where it was noted that ‘the low grade mental defectives are not catered for at all [in specialist institutions], and they eventually drift into the mental hospitals. The resident medical superintendents only take them as there is nowhere else to send them.’^43^ Clearly, mid-century medical professionals remained tied to this established admissions practice, regardless of their increased autonomy within this legislation.

As ‘handicap admissions’ persisted, living conditions continued to decline within psychiatric hospitals across the country. Life within the district asylum system had been frequently poor during the nineteenth century, where overcrowding of wards was a regular complaint.^44^ This issue persisted into the twentieth century, as inspection reports outlined the clearly overcrowded living conditions across the state’s (increasingly dilapidated) Victorian hospital infrastructure. A 1951 Inspection of Enniscorthy described how overcrowding ‘extended to every department of the hospital’, while units in Grangegorman were ‘extremely overcrowded’, and in Ardee ‘overcrowding was evident on both sides of this institution … in some dormitories it was necessary to include an extra row of beds each night which had to be removed each morning’.^45^ To an extent, these practices were inevitable given the considerable populations that were housed within these facilities. While the rate of psychiatric institutionalisation had remained high throughout the nineteenth century, this population continued to expand into the mid-twentieth century, peaking in 1958 at 21,075 in-patients or 0.7 per cent of the state’s total population.^46^ This pressure on space influenced daily life within these hospitals. The twin concerns of overcrowding and poor living conditions were vividly outlined in Browne’s memoir, which recounted his first visit to Grangegorman (St. Brendan’s), the largest psychiatric hospital in the state, in 1959. Overcrowding was then endemic across the hospital, as ‘many of the wards at that time had upwards of a hundred patients in them’. Understandably, this fostered less than ideal conditions for both staff and residents. Browne described how, on the evening of his first visit:

there were crowds of patients all jostling each other, some of the women with their dresses pulled up over their heads and here and there a nurse, struggling amid the chaos. There was a cacophony of sound and I felt as though I was lost in some kind of hell.^47^

Nationwide, the in-patient population began to decline from 1958, although reliance on ‘mental hospital’ beds remained notably high when compared against European norms into the 1970s.^48^ In 1962, as a senior medical officer, Browne was transferred to Portrane (St. Ita’s) hospital in North Dublin. He encountered similar levels of overcrowding and described how this contributed to the marginalisation of the centre’s intellectually disabled patients, as:

St. Ita’s [was] even more depressing than St. Brendan’s, with the long, sombre corridors leading to large wards full of forlorn human beings … There were old dilapidated huts where the most disabled of the mentally retarded were housed. These were known as the wet and dirty wards, full of small, gnome-like creatures in long black coats sitting and standing around on floors impregnated with years of urine.^49^

This unsafe and unsanitary accommodation, originally built as temporary housing for builders at the hospital in the early twentieth century, remained in use until 1982 when the huts’ floors partially collapsed.^50^ Browne acknowledged that this deficient treatment was not consistent across all psychiatric institutions in the state. St. Loman’s in Palmerstown, for instance, was a ‘small, clean, well run hospital with a buoyant, optimistic atmosphere’.^51^

Yet, departmental reports indicate that St. Loman’s was an exception across the broader field of psychiatric services in the mid-century, as ‘handicapped patients’ continued to enter obviously unsuitable institutional environments throughout this period. A 1959 memorandum for John Brady, assistant secretary at the Department of Health, warned of the effect of overcrowding on vulnerable patients like the disabled in psychiatric facilities, describing how ‘we are keeping patients at a low level of animal existence and actively destroying any bit of individuality, confidence or self-respect the may have left’.^52^ The Assistant Inspector of Mental Hospitals echoed this assessment and bemoaned how, regardless of the successes in a centre like St. Loman’s, there was ‘no way of achieving coordination or organisation’ across different institutions.^53^ This meant that, although St. Loman’s in Dublin could be described as a great success and a model for patient care, just 43 miles away the psychiatric hospital in Mullingar (also called St. Lomans) served as an exemplar of the ‘ferocious institutionalised cruelty of the Irish mental hospital’.^54^ Considered together, these deficient living conditions clearly shaped the lives of ‘handicapped’ residents. In a report to the Dublin Health Authority in 1966, for instance, Ivor Browne described a system that could only provide basic physical care to its patients and was largely characterised by its ‘therapeutic inactivity, a low state of morale and an atmosphere not generally conducive to recovery’ for the acutely mentally ill, to say nothing of the impact on a long-term inpatient population like the intellectually disabled.^55^

By the mid-1960s, statutory disability policy was clear that psychiatric hospitals were ‘not suitable places for the mentally handicapped’.^56^ Yet, their admission to these centres was an established practice that can be traced back to the foundation of the district asylum system. Hospital records demonstrate the obvious reluctance of both doctors and governing boards to admit the disabled, as they acknowledged the deficiencies of a ‘mental hospital’ as housing for these vulnerable residents. Yet, the practice persisted due to a lack of alternative support, reflecting gaps in social policy within a state that continued to rely on a ‘mixed economy of welfare’ to address vulnerable groups like the disabled.^57^ However, notwithstanding the long history of this practice, shifts to the patient demographics within these facilities began to throw additional light on the intellectually disabled and their life within the state’s ‘mental hospitals’ during the mid-to-late twentieth century.

## ‘Still Getting Mentally Handicapped People’: Psychiatric Institutions, 1965–84

Speaking in 1991, Ivor Browne, the then Chief Psychiatrist of the Eastern Health Board, offered a narrative of progress to the Mental Health Association of Ireland when he discussed the (seemingly) inexorable decline of institutional psychiatric facilities.^58^ Admittedly, the transition away from congregate care was striking in this period, as the population of Grangegorman (St. Brendan’s) in inner-city Dublin fell from approximately 2,000 patients in 1960 to just over 300 in 1991.^59^ This decline in hospital in-patient populations, although extreme in Grangegorman’s case, was broadly replicated across the country, as the national inpatient psychiatric population fell from 21,720 in 1956 to 7,334 by 1990.^60^ Journalists also highlighted parallel changes to the approach within these services, as new treatment methodologies contributed towards an organisational culture where success was now counted ‘in terms of empty hospital beds’.^61^ For Browne, these changes were inevitable. His 1991 speech declared how efforts ‘in the Eastern region, [meant that] the demise of the mental hospital is nearly complete’.^62^ Yet, this contraction in patient numbers was not consistent across the different groups resident in the psychiatric system. The Commission on Mental Handicap highlighted how there were 2,594 intellectually disabled patients within ‘mental hospitals’ in 1965, which by 1981 had reduced by just 16 per cent to 2,170.^63^ By contrast, from 1963 to 1981, the number of hospital inpatients across the other major diagnostic categories fell at more than twice this rate; with a 48 per cent reduction in cases of ‘organic psychosis’, 41 per cent among Schizophrenics and 40 per cent among Manic-Depressives.^64^

The introduction of new psychopharmacological ‘treatments’ was crucial to these demographic shifts in Irish ‘mental hospitals’. Antipsychotics like chlorpromazine (Largactil), while not a cure for mental illness, now made ‘it possible for patients to tolerate their disorders with less anxiety and agitation’ through the minimisation of their physiological symptoms. Largactil was first trialled alongside the antipsychotic Reserpine (Serpasil) in Grangegorman in 1956. The trial’s results were very positive, so much so that the hospital’s Chief RMS Dr John Dunne described the new medication as a ‘now essential’ tool for psychiatric treatment. By reducing a patient’s acute symptoms, this medication helped to improve living conditions within a hospital, limiting incidents of violence and minimising the need for physical restraints. It also shortened the length of an average in-patient stay, making rapid discharges from the hospital increasingly possible. In 1965, 22.5 per cent of patients departed these centres within a month, by 1971 this had nearly doubled to 44.5 per cent.^65^

The impact of this medication was augmented by the propagation of out-patient psychiatric services. First used in the Adelaide hospital in Dublin city in the 1930s, the availability of out-patient clinics grew considerably throughout the late 1950s and into the early 1960s.^66^ In 1957, St. Loman’s in Mullingar had 3,491 outpatient attendances, by 1962 this had increased to 13,340 annually.^67^ Ivor Browne established the first psychiatric clinic in the Dublin suburb of Ballyfermot in the early 1960s. He described encounters with patients who faced an array of ‘virtually insoluble social problems … who were depressed, anxious, overwhelmed and ready to give up’.^68^ Regardless of the severity of their issues, this community-based service allowed Browne to treat patients without requiring their admission to an institution; he could change ‘the prescriptions from one antidepressant or tranquiliser to another’ while they continued to reside in the wider community.^69^ Taken together, out-patient treatment and new psychopharmacological medications catalysed the sharp decline in certain patient groups within the psychiatric hospital system. Yet, these innovations had little impact on discharges for the ‘mentally handicapped’ in-patient.

Reports from visiting committees, and accounts provided by hospital medical staff, highlight that the admission of intellectually disabled patients to psychiatric facilities continued to precipitate overt disquiet in the period between 1965 and 1984, but persisted due to a chronic lack of alternative forms of institutional accommodation. The visiting committee at St. Finan’s Hospital in Killarney was informed that the institution’s 804 patients in 1965 represented ‘the lowest population figure in the hospital for over 10 years’. However, despite this reduction in the overall patient population, the hospital’s RMS Dr J.J. O’Connor expressed concern about how ‘they were still getting Mentally Handicapped people into the hospital, both children and adults’ despite the doctor’s reservations regarding the standard of care available in the facility.^70^ In a response to Dr O’Connor, Kerry County Manager P. O’Halloran accounted for the continued need to send the ‘handicapped’ to St. Finans. He blamed the practice on the discharge policies of nearby specialist residential centres, as these ‘institutions [will] only keep the children until they are 16’. Their approach, alongside a lack of services to support the disabled in the community, meant that the psychiatric hospital remained as the institutional provider of last resort.^71^

Indeed, admissions persisted in response to deficiencies across the broader disability services landscape. For instance, psychiatric facilities were still used when families were confronted with the (sizeable) waiting lists for specialist residential accommodation. The lack of places in disability-specific institutions drove a decision by the Cork Mental Health Authority in 1967, where it was agreed that in future ‘mentally defective’ adults would be placed in St. Raphael’s psychiatric hospital in Youghal.^72^ This consolidated the board’s previously *ad* *hoc* practice of moving disabled adults out of specialist facilities, like the Brothers of Charity centre at Lota, when they ‘were over age and could not be handled’;^73^ by the mid-1960s this approach had created a population of 136 ‘handicapped’ patients at St. Raphael’s.^74^ The North-West Health Board produced a similar proposal around the use of its psychiatric facilities in 1972, recommending that handicapped adults were transferred to ‘mental hospitals’ as a way to free up residential beds for children.^75^ This measure was undesirable but necessary, the board argued, as ‘a significant proportion’ of those on lists for residential centres had been waiting for more than 6 years.^76^

In a similar vein, multiple regional health authorities planned specialist wards for the disabled within existing ‘mental hospital’ facilities. In 1968, St. Brigid’s in Ballinasloe proposed an 18-bed ward for children whose ‘presence in the family home was causing undue hardship to the parents’ but who could not secure a residential placement.^77^ This measure was viewed as an imperfect solution to the broader challenges within an overtaxed system of residential provision. The Chairman of the Galway-Mayo Hospital board Senator Mark Killalea acknowledged the many issues associated with this approach, but nonetheless hoped that other psychiatric facilities would soon ‘follow their example’ in using the existing capacity of local ‘mental hospitals’ to provide specialist wards that could help necessitous families.^78^ A similar scheme was also discussed in 1972 for St. Columba’s in Sligo. Ray McSharry, the local TD (member of parliament), described the plan to accommodate disabled children in the hospital as a distasteful but necessary measure; these children had to be accommodated somewhere, he argued, especially before their ‘parents became psychiatric cases’ themselves from the stresses associated with home-based care.^79^ There were also plans to establish a unit for ‘handicapped’ boys on the grounds of St. Joseph’s Hospital in Limerick in 1968, although in this case the effort encountered strenuous resistance from that hospital’s RMS, Dr Niall O’Higgins. Speaking to the local health authority, Dr O’Higgins described the hospital as ‘the most unsuitable site imaginable’ and argued that the proposal ‘should create feelings of revulsion at the very thought’.^80^ In this case, his objections proved fatal to the project, with the effort abandoned in favour of developing services in collaboration with the Sisters of Saint Vincent de Paul.^81^

Dr O’Higgins’ objections appear exceptional, however, as by the early 1970s the intellectually disabled remained a significant minority population across the nation’s psychiatric hospital network. In 1971, St. Conal’s in Letterkenny housed 132 intellectually disabled residents, which was a quarter of their overall population, while St. Loman’s in Mullingar had 244 ‘handicapped’ patients (26 per cent).^82^ Indeed, the number of disabled patients across the district mental hospital network remained notably consistent, as admissions due to disability were described by health authorities as ‘generally stable and predictable from year to year’.^83^ Thus, as the overall in-patient population declined, the ‘handicapped’ continued to arrive at the doors of their local ‘mental hospital’, joining an increasingly prominent minority population within these facilities.^84^

Over time this trend was reflected in hospital statistics; while other patient groups departed the hospital, the ‘mentally handicapped’ became a growing proportion of the facility’s overall population. In the late 1950s, there were 2,241 ‘handicapped’ in-patients, which represented 10.6 per cent of psychiatric in-patients.^85^ In 1963, 14.3 per cent of in-patient beds were occupied by those classified as intellectually disabled.^86^ By the 1971 census of psychiatric facilities, the ‘handicapped’ population had increased again to 16.8 per cent of in-patients, despite remaining relatively static at 2,638.^87^ This decreased only slightly during the 1970s, with the 2,170 disabled residents in 1981 accounting for 15.5 per cent of beds.^88^ Thus, it is clear that while the number of ‘handicapped’ in-patients remained relatively consistent between 1965–84, they were a minority population within a declining system of provision which, due to new treatment methods and the development of community-based psychiatric services, simply did not have to house the mentally ill at the same rate. The steady decline in overall hospital patients encouraged the continuation of these admissions, as local authorities used increasingly empty hospital wards as a means of housing the disabled. This was despite the concerns of doctors, and with seemingly little reflection on the suitability of this accommodation for the intellectually disabled resident.

## ‘A Total and Absolute Air of Institutionalisation’: Inside the ‘Handicap Wards’

In May 1982, the Eastern Health Board’s Psychiatric Services Review Committee assessed the region’s in-patient facilities. They detailed how the ‘forbidding surroundings of the traditional nineteenth century mental hospital’ continued to serve as housing for significant numbers of both ‘mental handicap’ and geriatric patients, before acknowledging a ‘continuing and accelerating deterioration’ in their physical condition.^89^ It also underlined the disquiet among staff due to repeated failures to reform these services, where a culture of ‘learned helplessness and dependency’ compounded their day-to-day challenges.^90^ It came to the damning conclusion that reform efforts had not emerged as ‘there were few votes to be obtained behind the walls of mental institutions whose patients lacked a political voice and constituted a disenfranchised forgettable minority who could be electorally ignored’.^91^

This 1982 Health Board evaluation contended that little had changed since the mid-twentieth century, as the ‘mentally handicapped’ were still admitted to psychiatric institutions that were ill-equipped to meet their needs. To examine the lives of patients within an institutional setting presents obvious challenges.^92^ These challenges are particularly acute in Ireland, where there are even substantial gaps within the state’s statutory reporting mechanisms for psychiatric facilities during the 1960s and 1970s.^93^ Notwithstanding these concerns, a sobering image of life for the intellectually disabled emerges from extant hospital records; one where significant numbers of the ‘handicapped’ resided within unsanitary and often clearly unsafe physical accommodation, while relatives and professional inspectors expressed grave concerns about their daily life in these ‘hospital’ settings.

A growing scepticism regarding the standard of care available to the disabled within psychiatric units emerged in the UK and USA from the mid-1960s. This increased concern metastasized under the weight of sociological investigations and journalistic exposés, which revealed the deficient living conditions and limited services available to a disabled person resident in psychiatric care. Emblematic of this trend was Pauline Morris’s *Put Away* (1969). An extensive sociological survey of British institutions noted the particularly poor physical conditions within units that were associated with, or attached to, psychiatric hospitals.^94^ These physical deficiencies were compounded by a lack of activities or rehabilitative training, which ensured the continued dependency of disabled residents.^95^ Similar concerns were also discussed in the influential Ely Hospital Inquiry Report. Instigated by nursing assistant Michael Pantelides, the Inquiry began due to accusations that staff in the psychiatric institution near Cardiff routinely carried out a range of ‘cruel ill treatment … [and] generally inhumane and threatening behaviour towards patients … [including] pilfering of food, clothing and other items’.^96^ First discussed in *The News of the World* in August 1967, the accusations led to the creation of an inquiry headed by Geoffrey Howe.^97^ Alongside staffing failures and physical issues within the hospital, the inquiry report in 1969 also noted the significant population of ‘subnormal’ patients accommodated in Ely, who occupied 461 (75 per cent) of the hospital’s 614 beds.^98^ Their conclusions went on to have a long-lasting impact on British social policy concerning long-stay institutions for the intellectually disabled, prompting the White Paper *Better Services for the Mentally Handicapped* (1971) and the start of routine institutional inspections.^99^

A similar ‘uncovering’ of institutional deficiencies also occurred in the USA. Publications like Blatt and Kaplan’s *Christmas in Purgatory: A Photographic Essay on Mental Retardation* (1966), spotlighted copious failures associated with psychiatric facilities that housed the intellectually disabled. Blatt and Kaplan visited four large institutions in New England, using an early form of hidden camera. This resulted in a book that combined undercover photographs and detailed descriptions of life in this ‘land of the living dead’.^100^ *Christmas* described poor physical conditions within each institution, which commonly included: ‘gaping holes in ceilings’, fecal matter on walls, children left to crawl on exposed floors, as well as ‘rows and rows of benches on which sat countless human beings, in silent rooms waiting for dinner call or bedtime’.^101^ As it presented the reader with images of ‘bedlam, filth and runaway chaos’, Blatt and Kaplan’s research underscored the grave problems associated with retaining the disabled in an ill-equipped institutional setting, like a psychiatric facility, for a prolonged period of time.^102^

There was limited Irish press coverage of these UK and US investigations. Nonetheless, from the early 1970s, investigations into the state’s mental hospital network began to match these international counterparts by highlighting poor conditions within individual institutions and emphasising deficiencies in their care of vulnerable residents. One of the first clear manifestations of this new willingness to openly question the quality of institutional care was the publication of Hanna Greally’s memoir *Bird’s Nest Soup* (1971), a highly critical account of ‘mental hospitals’ in the mid-twentieth century. Charting her committal to St. Loman’s in Mullingar from 1943 to 1962, the book offered a rare glimpse into the largely obscured perspective of the psychiatric in-patient. And greally outlined obvious failures across the facility, including its poor living conditions, a lack of privacy and inadequate food.^103^ Alongside complaints about life in St. Loman’s, the memoir also underscored the continued ‘mixed’ nature of the hospital, which housed the ‘mentally ill but also … the poor, the eccentric, the socially troublesome, the vulnerable and the unwanted’ of Irish society.^104^

Aside from Greally’s exceptional account of life as a patient, there was also a growing body of press coverage that investigated poor living conditions within individual institutions. During a 1971 strike by psychiatric nurses, John Maddock from the *Evening Herald* detailed the ‘harrowing reports coming in from all over the country of the experiences in the hospitals’.^105^ Another *Evening Herald* reporter described the conditions in the ‘temporary’ hut accommodation at Portrane that housed the disabled, declaring that it was ‘so appalling that they bring shame on our health services’.^106^ Ivor Browne, who was then the programme manager for specialist hospital care at the Eastern Health Board, also increasingly spoke out in strong terms about the conditions within psychiatric institutions; he damningly concluded a 1972 report that the hospitals (which remained under his purview) were ‘substandard, antiquated and dehumanising’.^107^ These failures were brought into stark relief in November 1978, when 13 junior doctors publicly decried their working environment in St. Brendan’s (Grangegorman) in inner-city Dublin.^108^ Coverage of their initial complaint was followed by a series of longer profiles of the hospital, which presented a facility that was a ‘dickensian world of a badly lit passage maze connecting three floors of gloomy wards’.^109^ Journalists found numerous issues during their visits to the hospital, which included: ‘black and peeling’ walls, visible bird ‘droppings’ in the kitchens, a rodent problem (which the hospital management described as at ‘an acceptable level’) and antiquated sanitary facilities.^110^ Despite the Minister’s assurance that the hospital would close, these concerns persisted into the 1980s. In *Magill* magazine, journalist Helen Connolly described the degrading condition of Grangegorman’s residents and the potential dangers associated with daily life in one of these physically ailing wards. In 1980, for instance, she noted how a portion of the hospital roof fell on a sleeping patient. This revealed dry rot, but the ward continued to be used as accommodation due to a lack of space elsewhere in the unit.^111^

Portrane (St. Ita’s) in North Dublin housed the largest population of intellectually disabled patients within a psychiatric facility in the state; with approximately 500 ‘handicapped’ patients (42 per cent) among the hospital’s total population of 1,190 in 1977.^112^ In 1981, two detailed independent inspections were carried out of the ‘handicap’ wards in Portrane. The first was by Clare Kelly, from the Association of Parents and Friends of St. Ita’s, in February 1981. Kelly’s report provided a detailed account of the physically unsafe and unhygienic conditions across the hospital. In the Number 7 East ward for young ‘handicapped’ males, for instance, Kelly described an overwhelming ‘stench of urine’ across a ward that had soiled walls, where patients’ clothes were ‘ill fitting and dirty’, bedsheets were heavily stained and adorned with ‘rag-like’ blankets. Kelly offered a lengthy description of the ward’s bathroom, which was:

Scandalous. Two toilets. Behind those toilets there is a channel. Over on the right hand side looking in at the channel it is filled with a sludge-like matter. The shore is at the end of the channel, it is blocked and uncovered. It overflows all over the floor and patients must walk in this mess to use the toilets … Nurses and other staff say nothing is ever done about this.^113^

Her report found similar conditions in other ‘handicap wards’. In one that accommodated 35 patients, there was poor clothing, stained walls and floors, blocked toilets and a bathroom where ‘A sink was removed from the wall and never replaced. Two iron stays are [left] sticking from the wall.’^114^ In the Number 4 Male Ward, the report described a bleak environment, with broken floor tiles, blocked or overflowing toilets and excrement smeared walls. Kelly then went on to discuss some of the patients within these units, describing how one man sat on the floor with his feet touching effluent from the bathroom. This ‘unfortunate patient’ was left ‘just rocking to and fro’.^115^ Such conditions prompted her to conclude that talk of maintenance in the hospital was ‘a sick joke’, given that ‘only a blind man could ignore so many things needing urgent attention’ across the institution’s ‘handicap wards’.^116^

In June 1981, Portrane also received an inspection from representatives of the Irish Nursing Board (An Bord Altranais). Their visit had been requested by Ted Keyes, Programme Manager for Special Hospital Care in the Eastern Health Board, who asked the inspectors to examine the feasibility of providing segregated services for the hospital’s ‘mentally handicapped’ and psychiatric patient populations. Carried out between 3 and 5 June, the inspection report was an extensive litany of strongly worded complaints about the hospital’s poor physical condition, and the impact of this deficient living environment on its vulnerable patient population. In the children’s unit, where the majority of patients was classed as ‘severely mentally handicapped’, there was criticism regarding the lack of rehabilitative services, as inspectors noted how intellectually disabled children were ‘deprived of the multitude of therapeutic advances … afforded [to] other, equally handicapped but more fortunate children’.^117^ This neglect was contrasted against institutional facilities for the physically disabled, where patients were treated in ‘newly built, well equipped, interdisciplinary staffed hospitals, with a nursing complement of personnel, trained in the specific field of paediatric nursing’.^118^

Alongside a lack of therapeutic supports, there were consistent complaints regarding the physical condition and hygiene within wards. Ward 9A housed 30 ‘mentally handicapped’ patients and had a pervasive smell of urine throughout. The conditions in ‘Ward 4, Male’ were described as ‘nothing short of disgusting’, while ‘7 East’ was ‘bleak, utilitarian and absolutely non-stimulating’, which created an impression of ‘gloomy despondency’.^119^ These physical deficiencies had an impact on patients, the report argued, as there was a ‘total and absolute air of institutionalisation in Ward 5’, while in another ‘non-ambulant patients … just sit in their chairs around by the walls all day, doing nothing, saying nothing but thinking who knows what’. The inspectors even expanded their concern to address the professional well-being of staff members who worked in the facility, describing how: ‘As presently organised, functioning and existing, the student psychiatric nurse of 1981 had nothing beneficial, good or positive to learn in a hell hole like this. The unit is an affront to the dignity of man.’^120^ Following their visit, there was a meeting between hospital management and the Nursing Board’s Inspectors, during which the hospital’s RMS Dr Conway defended their standards by emphasising how the hospital was originally an annex of (the larger) Grangegorman Hospital, and that historically it ‘took the offal’ in terms of patients. Evidently, such an explanation was unsatisfactory, as the Nursing Board’s final report concluded with the damning summation that: ‘A lot of good men must have conspired over the years to do nothing, to have allowed St. Ita’s to deteriorate to its present condition.’^121^

The shock exhibited by these inspections raises an obvious question—why were conditions so poor in these ‘handicap wards’? This approach did not align with contemporary textbooks, like the eleventh edition of *Tredgold’s Mental Retardation* (1970), which emphasised the importance of hygiene and how ‘even the most severely subnormal children … [can be kept] clean and dry under supervision’.^122^ Instead the poor conditions in these wards were reflective of broader pressures on the psychiatric system in this period, including ever-tightening budgets as their in-patient population declined. In March 1980, Denis Dudley, CEO of the Southern Health Board, described the need to reduce expenses in his area. This entailed cuts to ‘additional expenditure’, which Dudley listed as ‘spending on furniture, crockery, bedding, clothing, heating, lighting, medicines, medical appliances, x-ray, pathology, travelling expenses, stationery and telephones’, leaving little that could be considered as essential for the operation of a residential institution.^123^ This contributed to the degraded conditions across multiple psychiatric institutions, including the disrepair encountered by both Clare Kelly and the Irish nursing board. Alongside budgetary pressures, the disabled represented a particularly vulnerable group within the mixed population of the psychiatric hospital, as they were unlikely to be able to articulate failures in their accommodation. This was highlighted during an RTÉ investigation in 1985, which noted how the majority of the disabled in psychiatric ‘handicap wards’ were non-verbal. Mary’s son Danny, for instance, resided in the huts in Portrane. She noted how Danny was an exceptional patient in this setting as he could speak, while others ‘were ill-treated, they all were but there was only a few patients on the ward who could talk’.^124^ ‘Handicap admissions’ could therefore be placed in the most degraded wards of a hospital where they were subject to little more than basic physical care. Yet, parents of these residents were unlikely to complain about these conditions. Disability activist Annie Ryan explained that this group was particularly vulnerable as ‘when they’re adults often [they] don’t have relatives … some of them are abandoned and they certainly can’t protest on their own behalf’.^125^

## Conclusion

An established practice can be hard to break. In January 2018, the psychiatric system was again used to accommodate a vulnerable intellectually disabled man, described by the President of the High Court Justice Peter Kelly as ‘seriously underweight, incontinent and at risk of sepsis’.^126^ Although it was deemed highly inappropriate, the psychiatric hospital remained the accommodation provider of last resort, perpetuating an admissions practice that can be traced back to the foundation of the asylum system in the early nineteenth century. Media coverage in 2018 noted how this young man should not be placed in a psychiatric facility, particularly when ‘several reports stated he is not psychiatrically ill’.^127^ However, this made him no different from the thousands of intellectually disabled admissions to ‘mental hospitals’ throughout the twentieth century.

Heretofore, research on the ‘deinstitutionalisation’ of Ireland’s psychiatric hospitals has resulted in a policy-based narrative that charts the steady decline of their inpatient population, which fell by more than 60 per cent from 1965 to 1990.^128^ Yet, this approach elides those who were the last to depart these ailing and ill-equipped hospital facilities, neglecting their experiences and producing a simplified transition from institutional to community-based psychiatry. To be clear, throughout the twentieth century, ‘handicap admissions’ to psychiatric institutions were a minority, as most of the intellectually disabled resided in the community. In 1904, the Royal Commission on the Care and Control of the Feeble-Minded estimated that there were at least 25,000 ‘mental defectives’ across the country, while at a 1938 meeting of the Statistical and Social Inquiry Society of Ireland, Dr Louis Clifford proposed that there were potentially thousands of ‘mentally deficient schoolchildren’ unknown to Dublin’s education authorities.^129^ By 1960, the *Problem of the Mentally Handicapped* White Paper considered that there were at least 24,000 people with a ‘mental handicap’ outside of residential care, 7,000 of whom were deemed to require some form of permanent institutionalisation.^130^ By the late-1970s the take-up of social welfare payments like the Disabled Persons’ Maintenance Allowance, alongside the work of Dr Michael Mulcahy for the Medico-Social Research Board, gave these previously nebulous estimates a more concrete basis, pointing to a considerable disabled population of approximately 14,658 who lived in the wider community.^131^

Yet, despite being a minority of the intellectually disabled overall, the experiences of those within institutional psychiatric care presents insights that can contribute towards Ireland’s disability history more broadly. At a basic level, the persistence of their admission to these ailing institutions reveals the limited capacity of specialist institutional services. Hospital governing boards consistently noted their reluctance to admit the ‘mentally handicapped’ but continued to do so due to a lack of alternative measures. Psychiatric institutions reacted to the need for secure accommodation, regardless of how inappropriate their response was in practice. These admissions practices also exhibit the limits of legislative reforms like the 1945 *Mental Treatment Act*; which, despite consolidating power in the hands of physicians, had a negligible impact due to the lack of ancillary supports and the public expectation that the necessitous applicant had to be admitted somewhere.

By exploring the experiences of the intellectually disabled within psychiatric institutions, this article has sought to begin the investigation of both disability and psychiatric care in post-war Ireland. The persistence of the disabled in these facilities complicates the existing narrative of steady decline, while an analysis of provision for the disabled contributes towards the broader histories of institutionalisation, social security and social policy. The experiences of the disabled admitted to these institutions represent a singular avenue of enquiry in the state’s still nascent ‘disability history’, much more research is required if we are to fully comprehend the provision landscape available to the ‘mentally handicapped’ person in Ireland during the late twentieth century.

## Acknowledgements

I would like to thank the anonymous reviewers for their thought provoking advice and feedback regarding this article. Thanks are also due to Dr. Kevin O'Sullivan and Dr. Sarah-Anne Buckley for their support. Thanks also to Margaret Hurley of the Wellcome Trust for her assistance with bringing this article into public. The research for this article was only possible through the support of the Wellcome Trust (PhD Award, 108597/Z/15/Z). 

**Conflict of interest statement**: none declared.

## Footnotes

1 This article uses the term intellectual disability to denote a broad range of conditions that have been associated historically with forms of cognitive or functional impairment. The term is used in light of its prevalence in Ireland, while ‘learning disability’ and ‘developmental disability’ remain the dominant nomenclature in the UK and USA, respectively. It also presents language that is clearly offensive, including terms like ‘mental handicap’, ‘mentally deficient’ and ‘retarded’. Their use is not intended to offend the reader or to condone this limited conception of the individual. Instead these terms are deployed to accurately present the linguistic discourse that surrounded intellectual disability, which grants an insight into how those with a disability were discussed throughout the mid-twentieth century.

2 Hilary Orpen, *Today Tonight No. 289*, RTÉ Archive, 13 August 1982.

3 *Ibid*.

4 See the discussion in—‘It’s Hard to Live on £145 Basic’, *Irish Press*, 23 August 1982, 7; Seamus Counihan, ‘Rescue Drive for ‘Forgotten’ in Hospitals’, *Evening Herald*, 6 August 1982, 4; Tom Shiel, ‘Cuts Leave Mental Hostels Empty’, *Irish Independent*, 13 November 1982, 9.

5 Aileen O’Hare and Dermot Walsh, *Irish Psychiatric Hospitals and* *Units* *Census: 1981* (Dublin: Medico-Social Research Board, 1982), 12.

6 In an Irish context, see: Damien Brennan, ‘A Theoretical Explanation of Institution-based Mental Health Care in Ireland’, in Pauline M. Prior, ed., *Asylums, Mental Health Care and the Irish 1800-2010* (Dublin: Irish Academic Press, 2012), 287; Brendan D. Kelly, ‘Mental Health Law in Ireland, 1945 to 2001: Reformation and Renewal’, *Medico-Legal Journal*, 2008, 76, 68; June Levine, ‘Afterword’, in Hanna Greally, ed., *Bird’s Nest Soup* (Cork: Cork University Press, 2008), 130. On deinstitutionalisation internationally, see: Despo Kritsotaki, Vicky Long and Matthew Smith, eds, *Deinstitutionalisation and After: Post-War Psychiatry in the Western World* (Basingstoke: Palgrave Macmillan, 2016); Peter Barham, *Closing the Asylum. The Mental Patient in Modern Society* (London: Penguin, 1992). 

7 Examples include: Alice Mauger, *The* *Cost* *of* *Insanity* *in Nineteenth Century Ireland* (Abingdon: Routledge, 2017); Catherine Cox, *Negotiating Insanity in the Southeast of Ireland, 1820-1900* (Manchester: Manchester University Press, 2012); Elizabeth Malcolm, ‘"Ireland’s Crowded Madhouses”: The Institutional Confinement of the Insane in Nineteenth and Twentieth-Century Ireland’, in Roy Porter and David Wright, eds, *The Confinement of the Insane: International Perspectives, 1800–1965* (Cambridge: Cambridge University Press, 2003), 315–33; Joseph Reynolds, *Grangegorman: Psychiatric Care in Dublin Since 1815* (Dublin: Institute of Public Administration, 1992); Mark Finnane, *Insanity and the Insane in Post-Famine Ireland* (London: Croom Helm, 1981).

8 These institutions were predominantly developed during the nineteenth century, a notable exception was the psychiatric hospital in Ardee, Co. Louth. For further information, see: Lisa Butterly, ‘Institutional Reform in Mental Healthcare in Ireland: The Establishment of the Ardee Mental Hospital, 1933, in Its Historical Context’ (PhD thesis, National University of Ireland, Maynooth, 2014). Damien Brennan, *Irish Insanity: 1800-2000* (Abingdon: Routledge, 2013), 93–116; Brendan D. Kelly, *Hearing Voices: The History of Psychiatry in Ireland* (Kildare: Irish Academic Press, 2016), 210.

9 See Louise Hide, ‘In Plain Sight: Open Doors, Mixed-sex Wards and Sexual Abuse in English Psychiatric Hospitals, 1950s—Early 1990s’, *Social History of Medicine*, 2018, 31, 732–53; David May, ‘The Baldovan Institution Abuse Inquiry: A Forgotten Scandal’, *History of Psychiatry*, 2019, 30, 267–82.

10 Vicky Long, ‘"Heading Up a Blind Alley”? Scottish Psychiatric Hospitals in the Era of Deinstitutionalization’, *History of Psychiatry*, 2017, 28, 116; Joan Busfield, ‘Mental Illness’, in Roger Cooter and John Pickstone, eds, *Medicine in the Twentieth Century* (Amsterdam: Harwood Academic Publishers, 2000), 639.

11 An exception to this is Mary E. Daly’s pioneering article on the education of the intellectually disabled in the 1950s. Mary E. Daly, ‘“The Primary and Natural Educator”?: The Role of Parents in the Education of Their Children in Independent Ireland’, *Éire-Ireland*, 2009, 44, 194–217. Broader historiographical reviews are available in: Julie Anderson and Ana Carden-Coyne, ‘Enabling the Past: New Perspectives in the History of Disability’, *European Review of History*, 2007, 14, 447–57; Anne Borsay, ‘History and Disability Studies: Evolving Perspectives’, in Nick Watson, Alan Roulstone and Carol Thomas, eds, *Routledge Handbook of Disability Studies* (Abingdon: Routledge, 2012), 324–34; Catherine Kudlick, ‘Disability History: Why We Need Another “Other”’, *The American Historical Review*, 2003, 108, 763–93.

12 Jonathan Andrews, ‘Case Notes, Case Histories, and the Patient's Experience of Insanity at Gartnavel Royal Asylum, Glasgow, in the Nineteenth Century’, *Social History of Medicine*, 1998, 11, 255–81, at 255.

13 Examples of this approach include: Catharine Coleborne, *Madness in the Family: Insanity and* *Institutions* *in the Australasian Colonial World, 1860-1914* (London: Palgrave, 2009), 65–87; Akihito Suzuki, ‘Lunacy and Labouring Men: Narratives of Male Vulnerability in Mid-Victorian London’, in Roberta Bivins and John Pickstone, eds, *Medicine, Madness and Social History: Essays in* *Honour* *of Roy Porter* (Basingstoke: Palgrave Macmillan, 2007), 118–28; Keir Waddington, ‘Thinking Regionally: Narrative, the Medical Humanities and Region’, *Medical Humanities*, 2015, 41, 51–56; Frank Crompton, ‘Needs and Desires in the Care of Pauper Lunatics: Admissions to Worcester Asylum, 1852-72’, in Pamela Dale and Joseph Melling, eds, *Mental Illness and Learning Disability since 1850: Finding a Place for Mental Disorder in the United Kingdom* (Abingdon: Routledge, 2006), 46–65; David Wright, *Mental Disability in Victorian England: The Earlswood Asylum, 1847-1901* (Oxford: Clarendon Press, 2001).

14 See Jan Walmsley, ‘Life History Interviews with People with Learning Disabilities’, in Robert Perks and Alistair Thomson, eds, *The Oral History Reader* (London, 1998), 126–40; Dorothy Atkinson, ‘Bringing Lives into Focus: The Disabled Person’s Perspective’, in David May, ed., *Transition and Change in the Lives of People with Intellectual Disabilities* (London: Routledge, 2001), 157–77.

15 Roy Porter, ‘The Patient's View’, *Theory and* *Society*, 1985, 14, 192.

16 See: Dermot Walsh and Antoinette Daly, *Mental* *Illness* *in Ireland 1750 - 2002: Reflections on the Rise and Fall of Institutional Care* (Dublin: Health Research Board, 2004); Brennan, *Irish* Insanity..

17 Finnane, *Insanity*, 13.

18 Stephen J. Ball, *Foucault, Power, and Education* (Abingdon: Routledge, 2013), 66; Kelly, *Hearing Voices*, 54.

19 Mauger, *The Cost of* *Insanity*, 69; Cox, *Negotiating Insanity*, 170.

20 *Condition of the Poorer Classes in Ireland: First Report* (London, 1835), quoted in Joseph Robins, *Fools and the Mad: A History of the Insane in Ireland* (Dublin: Institute of Public Administration, 1986), 159.

21 Brennan, ‘A Theoretical Exploration’, 287; Finnane, *Insanity*, 34; Kelly, *Hearing Voices*, 70.

22 *Fifth Report of Lunacy Inspectors* (Dublin, 1851) quoted in Cox, *Negotiating* *Insanity*, 174–75.

23 *Commission on Relief of the Sick and Destitute Poor including the Insane Poor* (Dublin: Stationery Office, 1927), 101; Finnane, *Insanity*, 221.

24 Finnane, *Insanity*, 221.

25 Elizabeth Malcolm,‘‘Ireland’s Crowded Madhouses’: The Institutional Confinement of the Insane in Nineteenth and Twentieth-century Ireland’, in Porter and Wright, eds, *Confinement of the* *Insane*, 328; Hensey, *The Health Services of Ireland*, 25; Kelly, ‘Mental Health Law in Ireland’, 66.

26 Patricia Casey and Ciaran Craven, *Psychiatry and the* *Law* (Dublin: Oak Tree Press, 1999), 458.

27 Brendan D. Kelly, ‘The Mental Treatment Act in Ireland: An Historical Enquiry’, *History of Psychiatry*, 2008, 19, 47.

28 Vincent Dolphin, *Report of the Inspector of Mental Hospitals for the Year 1956* (Dublin: Stationery Office, 1957), 34; Tom Feeney, *Seán MacEntee*, 157.

29 Ivor W. Browne, *Music and* *Madness: A Personal Journey* (Cork: Cork University Press, 2008), 139.

30 A. Jamie Saris, ‘Mad Kings, Paper Houses, and an Asylum in Rural Ireland’, *American Anthropologist*, 1996, 98, 548.

31 Brendan D. Kelly, ‘Searching for the Patient's Voice in the Irish Asylums’, *Medical Humanities*, 2016, 42, 87–91, at 89.

32 Eilís Ward, ‘Foreword’, in Greally, *Bird's Nest Soup*, xiv.

33 ‘Grangegorman Mental Hospital Joint Committee Minutes’, 17 June 1948, 314, http://www.lenus.ie/hse/handle/10147/121530 (accessed 5 May 2016).

34 John Dunne, qtd. in ‘Grangegorman Mental Hospital Joint Committee Minutes’, 20 October 1949, 258, http://www.lenus.ie/hse/handle/10147/121531 (accessed 5 May 2016)

35 Dolphin, *Report 1956*, 63; Fiachra Byrne, ‘Madness and Mental Illness in Ireland: Discourses, People and Practices: 1900-60’ (PhD thesis, University College Dublin, 2011), 250.

36 Byrne, *ibid*., 252.

37 Dolphin, *Report 1956*, 34.

38 For further discussion of these challenges see: Brendan D. Kelly, ‘Intellectual Disability, Mental Illness and Offending Behaviour: Forensic Cases from Early Twentieth Century Ireland’, *Irish Journal of Medical Science*, 2010, 179, 409.

39 RMS Limerick to Inspector of Mental Hospitals (Joseph Kearney), 8 November 1946. Previously filed under NAI HLTH L50/2 Vol. 2. Viewed by private appointment at the Records Management Unit (hereafter NMU), Department of Health, Hawkins House, 5 April 2017.

40 *Ibid*.

41 *Ibid*. Institutions like Stewarts (Stewart’s Institute for Idiotic and Imbecile Children and Middle-Class Lunatics) and St. Augustine’s were private philanthropic facilities that housed populations of the intellectually disabled. The majority of these centres were operated by Catholic religious orders, while the state providing them with an annual grant to cover a proportion of their costs.

42 Annie Ryan, *Walls of Silence: Ireland's policy towards people with a mental disability* (Kilkenny: Red Lion Press, 1999), 41.

43 Anonymous, ‘The Problem of Mental Defectives’, *Kilkenny People*, 28 September 1957, 7.

44 Brennan, *Irish* *Insanity*, 99; Cox, *Negotiating* *Insanity*, 174; Finnane, *Insanity*, 54.

45 Dolphin, *Report of the Inspector of Mental Hospitals for the* *Year* *1951* (Dublin, 1952), 25; Dolphin, *Report of the Inspector of Mental Hospitals for the year 1952* (Dublin: Stationery Office, 1953), 23; Dolphin, *Report of the Inspector of Mental Hospitals for the* *Year* *1954* (Dublin: Stationery Office, 1955), 22.

46 Vincent Dolphin, *Report of the Inspector of Mental Hospitals for the Year 1959* (Dublin: Stationery Office, 1960), 20; Walsh and Daly, *Mental Illness in Ireland*, 33.

47 Browne, *Music and* *Madness*, 67.

48 Mary Daly, *Sixties Ireland: Reshaping the Economy, State and Society, 1957-1973* (Cambridge: Cambridge University Press, 2016), 248.

49 Browne, *Music and Madness*, 103.

50 Brendan O’Connor, ‘Hearing the Forgotten’, *Sunday Independent*, 30 May 1999, 17; Eddie Barrett, ‘Huts House Hospital Patients’, *RTÉ News*, 1 August 1978, RTÉ Archive Online, https://www.rte.ie/archives/2018/0720/979942-hunger-strike-for-portrane-patients (accessed 12 September 2018).

51 Browne, *Music and Madness*, 105.

52 Ciara Meehan, *A Just Society*, 46–47.

53 Christine Linehan *et al*., *Mapping the National Disability Policy Landscape* (Dublin: Trinity College, 2014), 2.

54 Ward, ‘Foreword’, xiv.

55 Ivor W. Browne, ‘Psychiatric Services’, in John O’Regan, ed., *Report of the Chief Medical Officer for the* *Year* *1966* (Dublin: Stationery Office, 1967), 132.

56 Commission on Mental Handicap, *Report of the Commission on Mental Handicap* (Dublin: Stationery Office, 1965), 111.

57 John Curry, *Irish Social Services* (Dublin: Institute of Public Administration, 2003), 177–87; Andrew Power, *Landscapes of Care: Comparative Perspectives on Family Caregiving* (Aldershot: Ashgate, 2010), 38; Bairbre Redmond and Anna Jennings, ‘Social Work and Intellectual Disability: A Historical Overview’, in Noreen Kearney and Caroline Skehill, eds, *Social Work in Ireland: Historical Perspectives* (Dublin: Institute of Public Administration, 2005), 107–27.

58 Erving Goffman, *Asylums: Essays on the Social Situation of Mental Patients and Other Inmates* (London: Anchor Books, 1968), 1.

59 Ivor W. Browne, ‘Community Care’, 325.

60 Kelly, ‘Mental Health Law in Ireland’, 68; Walsh and Daly, *Mental Illness in Ireland*, 69.

61 Levine, ‘Afterword’, 130, It should be noted that June Levine was married to Ivor Browne; Michael Viney, ‘Mental Illness, An Inquiry II: The Opening Door’, *The Irish Times*, 24 October 1963, 10; ‘Mental Hospitals to Be Phased Out Plan’, *Evening Herald*, 23 June 1972, 3.

62 Browne, ‘Community Care’, 325.

63 Commission on Mental Handicap, *Report 1965*, 108; Dermot Walsh and Aileen O’Hare, *Activities of Irish Psychiatric Hospitals and Units, 1981* (Dublin: Medico-Social Research Board, 1983), 12.

64 Walsh and Daly, *Mental Illness in Ireland*, 75.

65 Dermot Walsh and Aileen O’Hare, *Activities of Irish Psychiatric Hospitals and Units 1965-9* (Dublin: Medico-Social Research Board, 1970), 84; Walsh and O'Hare, *Activities of Irish Psychiatric Hospitals and Units 1971* (Dublin: Medico-Social Research Board, 1972), 33.

66 Kelly, *Hearing Voices*, 214–15.

67 Viney, ‘Mental Illness, an Inquiry II’, 10; Browne, ‘Psychiatric Services’, 152.

68 Browne, *Music and Madness*, 123.

69 *Ibid*., 123.

70 ‘Killarney Hospital Problem’, *Cork Examiner*, 3 September 1965, 18; ‘Lowest Number of Patients in Ten Years’, *Kerryman*, 4 September 1965, 7.

71
*Ibid.*

72 ‘Decision to Re-group Youghal patients’, *Southern Star*, 14 October 1967, 2.

73 ‘Proposed Transfer of Mental Defectives from Lota’, *Cork Examiner*, 29 May 1962, 6.

74 ‘Youghal Patients Transfer to Macroom’, *Southern Star*, 18 February 1967, 3.

75 ‘Facilities for Handicapped Boys’, *Sligo Champion*, 30 June 1972, 19.

76 ‘Health Board to Take Urgent Action to Accommodate Severely Handicapped Children’, *Donegal Democrat*, 24 November 1972, 17.

77 ‘Hospital Unit for Retarded Children Approved’, *Connacht Tribune*, 13 September 1968, 24.

78
*Ibid.*

79 ‘Discussions of Mentally Handicapped Children’, *Sligo Champion*, 27 October 1972, 11.

80 ‘RMS Opposes Minister’s Suggestion for Retarded Boys’, *Cork Examiner*, 9 November 1968, 11.

81 *Ibid*.

82 ‘Call for Psychiatric Social Workers’, *Donegal Democrat*, 5 November 1971, 14; Aileen O’Hare and Dermot Walsh, *Irish Psychiatric Hospital Census 1971* (Dublin: Medico-Social Research Board, 1971), 41.

83 Aileen O’Hare and Dermot Walsh, *Activities of Irish Psychiatric Hospitals & Units, 1973 & 1974* (Dublin: Medico-Social Research Board, 1975), 14.

84 Walsh & Daly, *Mental Illness* *in Ireland*, 75.

85 Joseph Dolphin, *Report of the Inspector of Mental Hospitals for the Year 1959* (Dublin: Stationery Office, 1959), 32.

86 Social Affairs Committee, *Psychiatric Services: Present Policies and Problems* (Dublin: Department of Health, 1978), 15.

87 Comhairle na nOspidéal, *Psychiatric Services at Consultant Level: A Discussion Document* (Dublin: Comhairle na nOspidéal, 1978), 20.

88 Aileen O’Hare and Dermot Walsh, *Activities … 1981*, 12.

89 Eastern Health Board, *Report of the Psychiatric Services Review Committee* (Dublin, May 1982), 7–8.

90 *Ibid*., 11.

91 *Ibid.*, 23.

92 Kelly, ‘Intellectual Disability, Mental Illness’, 412; Kelly, ‘Searching’, 87; Iain Hutchinson, ‘Institutionalization of Mentally Impaired Children in Scotland c.1855-1914’, *History of Psychiatry*, 2011, 22, 430; Ann Sheridan, ‘Being a Psychiatric Nurse in Ireland in the 1950s’, in Gerard M. Fealy (ed.), *Care to Remember: Nursing and Midwifery in Ireland* (Cork: Mercier Press, 2005), 176; Andrews, ‘Case Notes’, 237.

93 Despite a legislative obligation to do so, the National Inspector of Mental Hospitals did not publish a public report on psychiatric institutions for more than a decade from 1965, a lapse in oversight that remains unexplained. Ryan, *Walls of Silence*, 1–3.

94 Pauline Morris, *Put Away: A Sociological Study of Institutions for the Mentally Retarded* (London: Routledge and Kegan Paul, 1969), 120–21.

95 *Ibid*., 169, 281–82.

96 Michael Pantelides (cited as XY) in *Report of the Committee of Inquiry into Allegations of Ill-Treatment of Patients and Other Irregularities at the Ely Hospital, Cardiff* (London: HMSO, 1969), Sections 1–2.

97 Ian Butler and Mark Drakeford, *Scandal, Social Policy and Social Welfare* (Bristol: Policy Press, 2005), 43.

98 *Report* ... *at* *Ely Hospital, Cardiff*, sections 2–16.

99 Butler and Drakeford, *Scandal*, 59; Charles Webster, *The National Health Service: A Political History* (Oxford: Oxford University Press, 2002), 119; Niall McCrae and Peter Nolan, *The Story of Nursing in British Mental Hospitals: Echoes from the Corridors* (Abingdon: Routledge, 2016), 189; Jonathan Toms, ‘Mind the Gap: MIND, the Mental Hygiene Movement and the Trapdoor in Measurements of Intellect’, *Journal of Intellectual Disability Research*, 2010, 54, 16.

100 Burton Blatt and Fred Kaplan, *Christmas in Purgatory: A Photographic Essay on Mental Retardation*, 2nd edn (New York: Human Policy Press, 1974), 5.

101 *Ibid*., 1, 13, 47.

102 Robert Bogdan *et* *al*., *Picturing Disability: Beggar, Freak, Citizen and Other Photographic Rhetoric* (New York: Syracuse University Press, 2012), 73.

103 Greally, *Bird’s Nest Soup*, 1–42.

104 *Ibid*., 3–5; Ward, ‘Foreword’, xiii.

105 John Maddock, ‘Patients in Sorry Plight’, *Evening Herald*, 10 November 1971, 1.

106 ‘Hospital Patients in 90-year-old Wooden Huts’, *Evening Herald*, 7 September 1971, 7.

107 ‘Mental Hospitals to Be Phased Out Plan’, *Evening Herald*, 23 June 1972, 3.

108 Browne, *Music and Madness*, 147.

109 Michael Sharkey, ‘The Grim Wards of St. Brendan’s’, *Irish Press*, 14 November 1978, 1–3; Gerard O’Regan, ‘Rat Ridden Hospital Scandal’, *Irish Independent*, 10 November 1978, 1.

110 ‘Haughey Agrees that Hospital May Be as Bad as Doctors Say’, *Irish Press*, 11 November 1978, 3; ‘Not Good Enough’, *Irish Independent*, 16 November 1978, 10; ‘That Man Again’, *Western People*, 18 November 1978, 10; ‘A Five Year Wait?’, *Irish Press*, 13 December 1978, 8.

111 Helen Connolly, ‘The Scandal of the Mental Hospitals’, *Magill*, 30 October 1980.

112 Ivor Browne, *Development of Community Mental Health Services: Planned Evolution* (Dublin: Eastern Health Board, 1977), 68.

113 Clare Kelly, ‘Report of Visit to St. Ita’s, 11 February 1981’, ICA Papers, NLI MS 39,365.

114 *Ibid*.

115 *Ibid*.

116 *Ibid*.

117 R. McGuiness *et al.*, ‘Report on a 3 Day Inspection by a Select Committee of the Psychiatric Sub-committee of An Bord Altranais, Carried Out on the 3,4,5, June 1981’, An Bord Altranais Papers, UCDA P220/381, 9.

118 *Ibid*., 9.

119 *Ibid*., 11.

120 *Ibid*., 11.

121 *Ibid.*, 23.

122 Roger Francis Tredgold and Kenneth Soddy, *Tredgold's Mental Retardation: 11th Edition* (London: Baillière, Tindall and Cassell, 1970), 74–75.

123 Helen Connolly, ‘The Scandal of the Mental Hospitals’.

124 Mary, *Today Tonight No. 635*, RTÉ Archive, 21 February 1985.

125 Barrett, ‘Huts House Hospital Patients’*.*

126 ‘Young Man with Intellectual Disability to Be Discharged from Hospital into Community’, *The Irish Examiner*, 23 January 2018.

127 *Ibid*.

128 Antoinette Daly and Dermot Walsh, *Irish Psychiatric Units and Hospitals Census: 2006* (Dublin: Health Research Board, 2007), 19.

129 Louis S. Clifford, 'Investigation into the Incidence of Mental Deficiency Amongst Dublin School Children', *Journal of the Statistical and Social Inquiry Society of Ireland* 1939/1940, 16, 43–8

130 The 1965 Report of the Commission of Inquiry on Mental Handicap acknowledged the lack of information around the number of those with an intellectual disability living outside of institutional accommodation in Ireland. The report instead applied the prevalence rates used for the 1929 UK Report of the Mental Deficiency Committee (the Wood Report) to the population of 1960s Ireland. Commission of Inquiry on Mental Handicap, Report of the Commission of Inquiry on Mental Handicap (Dublin, 1965) 23–5.

131 *Medico-Social Research Board, Medico-Social Research Board: Annual Report 1983* (Dublin, 1984) 43.

## Notes

David Kilgannon is a PhD graduand of the National University of Ireland, Galway. His thesis, which was funded by the Wellcome Trust,explored changes to policy and provision for the intellectually disabled in twentieth century Ireland.

